# Rare Tumors of Oral Cavity: A Case Report and Literature Review on Secretory Carcinoma of Minor Salivary Glands

**DOI:** 10.1002/cre2.70200

**Published:** 2025-08-05

**Authors:** Emilio Salerno, Silvio Abati, Gianluigi Arrigoni, Daniela Finocchiaro, Giorgio Gastaldi, Alessandra Lissoni, Andrea Galli, Matteo Trimarchi

**Affiliations:** ^1^ Vita‐Salute San Raffaele University Milan Italy; ^2^ Department of Otolaryngology Head and Neck Surgery IRCCS San Raffaele Hospital Milan Italy; ^3^ Department of Dentistry and Stomatology IRCCS San Raffaele Scientific Hospital Milan Italy; ^4^ Department of Pathology IRCCS San Raffaele Scientific Hospital Milan Italy; ^5^ Department of Otolaryngology‐Head and Neck Surgery, Ente Ospedaliero Cantonale Ospedale Regionale di Lugano Lugano Switzerland; ^6^ Faculty of Biomedical Sciences Università della Svizzera italiana Lugano Switzerland

**Keywords:** genetics, mammary analogue secretory carcinoma, minor salivary glands, rare tumors

## Abstract

**Objectives:**

The primary objective of this study is to report and analyze two rare cases of secretory carcinoma (SC) of the minor salivary glands, focusing on their diagnostic and therapeutic work‐up. The study aims to enhance scientific knowledge about SC, which is crucial for developing targeted therapies and ensuring precise diagnosis, especially differentiating it from acinic cell carcinoma (ACC).

**Materials and Methods:**

The study involved a detailed examination of two patients diagnosed with SC of the minor salivary glands. Clinical examinations, histological investigations, and immunohistochemical analyses were conducted. The treatment approach included surgical excision of the lesions followed by regular follow‐ups to monitor for recurrence. Immunohistochemical analysis was performed to identify the presence of markers such as GATA3, SOX‐10, NTRK, mammaglobin, and others.

**Results:**

Two cases are shown: a case of a 76‐year‐old male with a lesion in the hard palate was initially misdiagnosed as leukoplakia. After surgical excision and histological examination, the lesion was identified as SC. The patient underwent follow‐up examinations, including MRI and CT scans, which showed no recurrence, and another case of a 39‐year‐old female with a nodule in the superior left vestibule underwent surgery to remove the nodule. Histological and immunohistochemical analyses confirmed SC, showing a high proliferation index and presence of the ETV6–NTRK3 gene fusion. Follow‐up imaging showed no signs of disease recurrence.

**Conclusions:**

The study underscores the importance of precise diagnosis and differentiation of SC from ACC. Surgical excision followed by regular monitoring is effective in managing SC. Immunohistochemical and molecular analyses are crucial for accurate diagnosis. The findings contribute to the growing body of evidence on SC and highlight the potential for developing more targeted therapies. Further research is needed to establish clear guidelines for follow‐up duration and treatment protocols.

## Summary

This article emphasizes how secretory carcinoma of salivary glands, which is not always indolent, should not be taken lightly when diagnosed. Specialized pathology knowledge is essential to understand the histopathological trait, leading to targeted therapy.

## 1 Introduction

Many research groups have focused their attention on a novel histological kind of salivary gland tumor, secretory carcinoma (SC), since the group of Skálová et al. (2010) recognized it as a stand‐alone variant of acinic cell carcinoma (ACC), with its own histological features. In many following investigations, several previous diagnoses were reallocated to this new entity thanks to the evidence of a fusion‐gene transcript. Chromosomal translocation t(12;15) (p13;q25) in SCs leads to an ETV6–NTRK3 gene fusion that is identical to that found in SC of the breast (Baghai et al. 2017; Tognon et al. 2002) and so this bring to N‐TRK nuclear immunohistochemical expression.

In 2017, the World Health Organization recognized it as a distinct item in the group of head and neck tumors, and, to ensure proper nomenclature, it was given the label of SC (Seethala and Stenman 2017).

This tumor shares histological and molecular similarities to SC of the breast, an uncommon malignancy that affects young people (Tavassoli and Norris 1980; Skálová et al. 2010). Indeed, the tissues of the breast and salivary glands come from the same ectodermal embryonic origin (Jackson et al. 2017). A slow‐growing painless mass, often appearing months or years before medical attention, is the most typical symptom (Sun et al. 2019; Chiosea et al. 2012). The predominance location of onset was studied in the major review of the literature on the issue at the moment (Silver et al. 2021): the parotid gland was found to have the highest incidence of SC (79.5%), followed by minor salivary glands (14.3%) and the submandibular gland (6.2%). SC of minor salivary glands arise most often in upper lip followed by buccal mucosa, hard palate, soft palate, and lower lip (Venkat et al. 2021). The male‐to‐female ratio was 1.5:1. These findings are similarly consistent with previous epidemiological findings from other studies (Skalova et al. 2017), but, if there appears to be a modest preference for males in Western countries, this data is contradicted by studies and reviews from Asian countries, which show no preference for either male or female gender (Ito et al. 2015; Jung et al. 2013).

The main differential diagnosis should be done with ACC, because clinically they are nearly similar, but their histological characteristics are quite different.

## 2 Cases Presentation

Overview of all the data pertaining to the patients taken into consideration is provided in the schematic table (Table 1).

**Table 1:** Schematic representation of patients' data.

.	Sex/Age	Localization	Clinical course	Treatment	Surgical margins	Status/Month
1	M/76	Hard left hemipalate	Mild	Surgical treatment	Negative	NED/19
2	F/39	Gingival mucosa of vestibule	Indolent	Surgical treatment	Close	NED/22

### 2.1 Case 1

#### 2.1.1 Chief Complaints and Physical Examination

**Figure 1:**
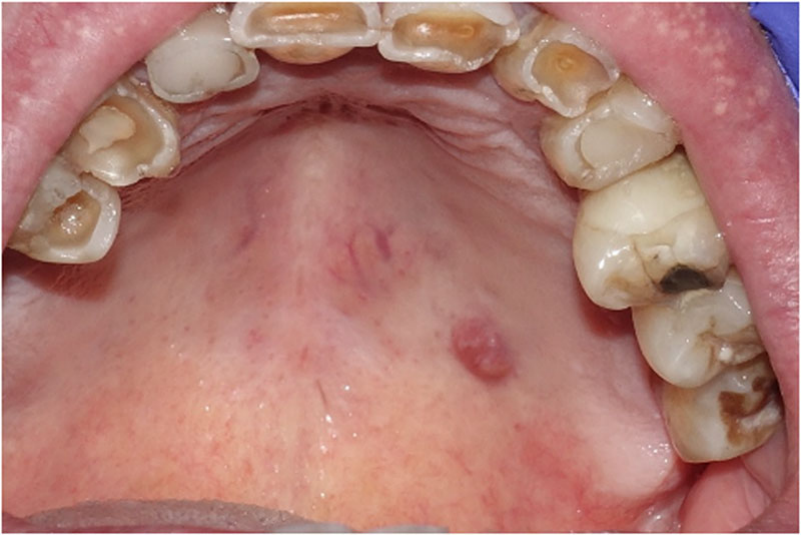
Patient 1 has a brownish formation in the hard hemipalate on the left side. This was not painful.

In July 2020, a general practitioner referred a 76‐year‐old male patient to San Raffaele's Dentistry unit for an indolent lesion of the hard palate that was misdiagnosed as “leukoplakia.” Clinical examination reveals a brownish‐purplish neoformation about 1 cm, in hard left hemipalate and it is consistent with a neoformation of a minor salivary gland or with pyogenic granuloma. The patient reports that this lesion has been there for many years without growing in size. When palpating the neck, there was no swelling to be found. There is no odontogenic pain from the dental components of the corresponding superior dental arch, and there is no pain on neoformation percussion. Given the benign suspicion regarding the lesion, resection of the lesion and histological investigation are advised (Figure 1).

#### 2.1.2 Treatment and Final Histological Diagnosis

**Figure 2:**
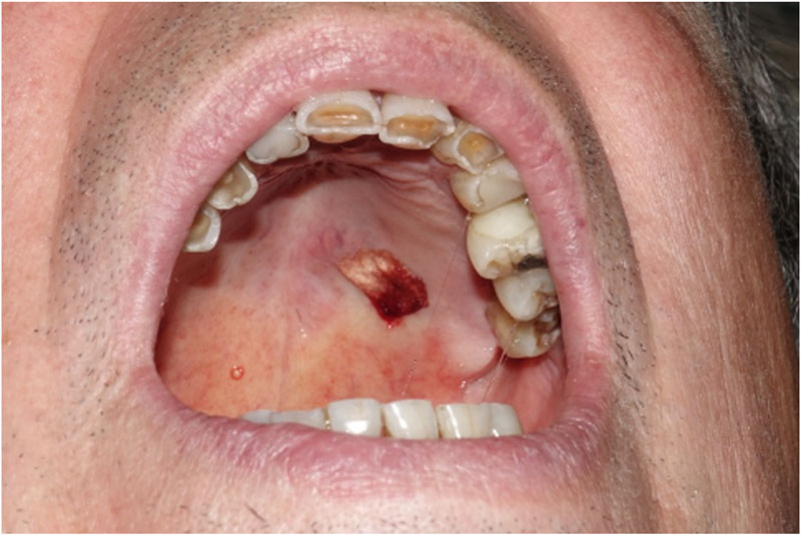
Patient 1. The picture shows the patient's condition following the lesion resection operation. The synthetic fabric utilized for hemostasis is outstanding.

**Figure 3:**
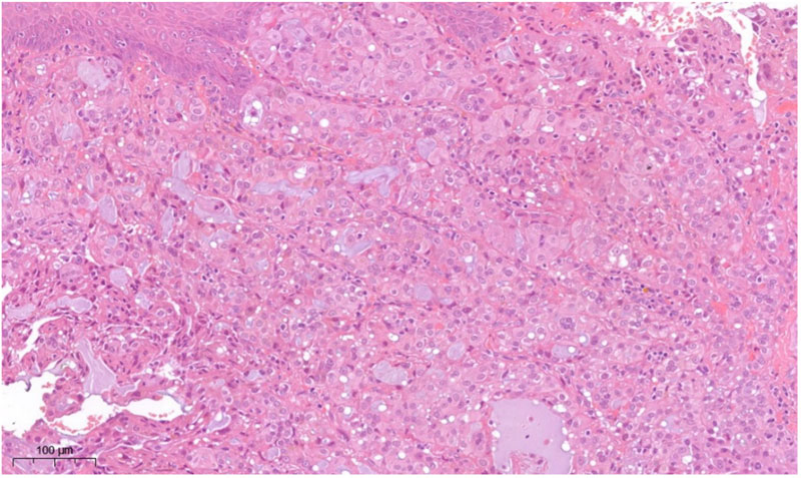
Tumor cells are arranged in solid areas separated by thin fibrous septa; tumor cells have round‐to‐oval nuclei with a small, centrally located nucleolus and eosinophilic cytoplasm (H&E, 20x).

In August 2020, the lesion was excised under local plexus anesthesia with 0.3 cc Articaine and vasoconstrictor, with hemostatic synthetic tissue for hemostasis (Figure 2). Safety margins of 4 mm were kept. Definitive histological examination indicates SC. Immunohistochemical analysis was performed: the neoplastic cells showed positivity for GATA3, SOX‐10, NTRK, mammaglobin, and negativity for actin, p40, GFAP, and CD117; the proliferation index was 5%, with margins free from neoplasm (Figure 3).

#### 2.1.3 Outcome and Follow‐Up

**Figure 4:**
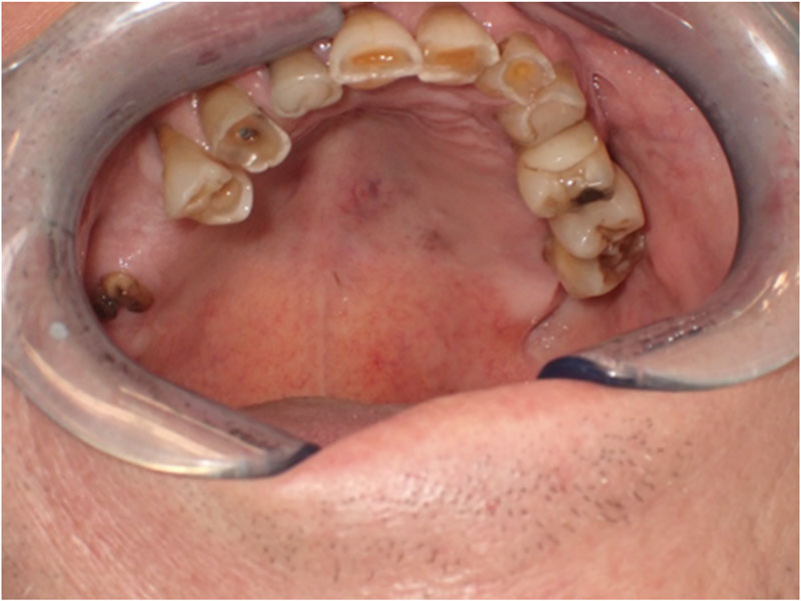
Patient 1. The picture shows the lesion after a year of observation. The fibrotic tissue that developed as a result of the procedure is visible.

Considering the findings of the final histopathological examination, the patient undergoes serial examinations in the year following surgery, including an mass facial MRI with contrast dye 1 month after surgery and a mass facial CT without contrast dye 3 months later. Throughout the follow‐up, both the instrumental control tests and the patient's clinical status are negative for illness recurrence. There are no changes to the palatal bone, no clinically or radiologically visible swellings, and no evidence of palpable lymphadenopathy. The patient received an ultrasound of the neck 3 months and 9 months afterward to monitor for lymphadenopathy; both tests were negative. One year after the procedure, a little swelling appeared at the surgical site, which was most likely due to scarring from the surgery. An examination for a potentially surgical intervention to treat this lesion is now ongoing (Figure 4).

### 2.2 Case 2

#### 2.2.1 Chief Complaints and Physical Examination

An indolent nodule in the superior left vestibule, below the oral mucosa, in the area of the canine‐premolar teeth, persisting, perceptible size less than 1 cm, in a 39‐year‐old female patient is brought to the attention of the Oral Pathology Department of Clinical of San Raffaele Hospital in May 2020. The patient reports that this lesion has been present for a few months without ever bleeding. The size has consistently been nearly constant. There was no swelling detected when the neck was palpated. Removing the lesion along with a histological examination are indicated considering the benign suspicion around it.

#### 2.2.2 Treatment and Final Histological Diagnosis

**Figure 5:**
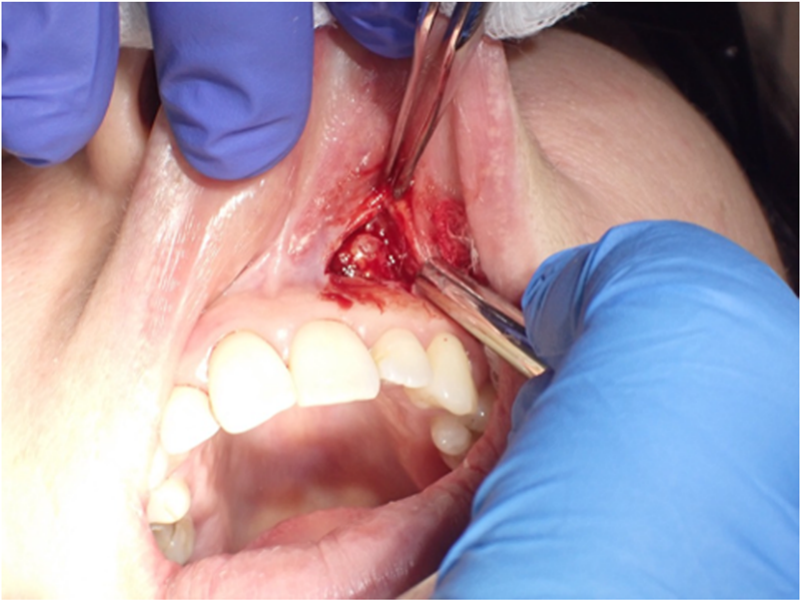
Photo of patient 2 taken during surgery. The lesion may be seen immediately below the gingival plane of the left vestibule, slightly below the mucosal plane.

**Figure 6:**
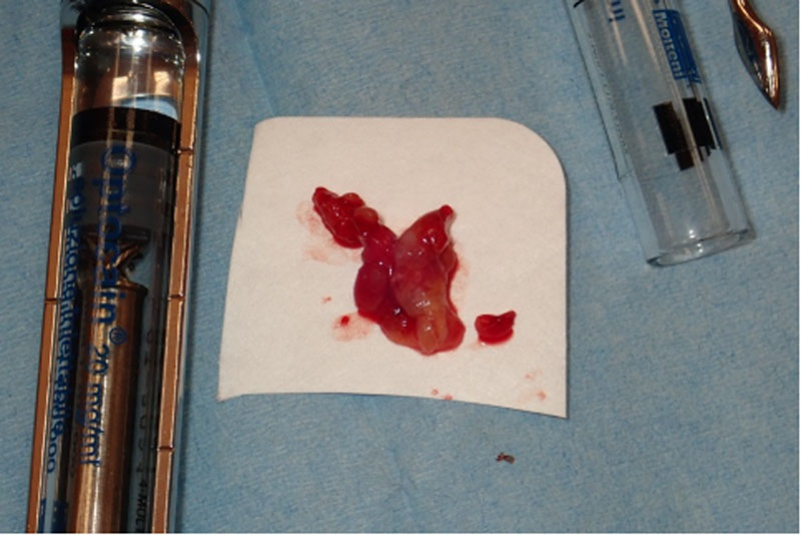
Patient 2. Surgical piece after removal.

**Figure 7:**
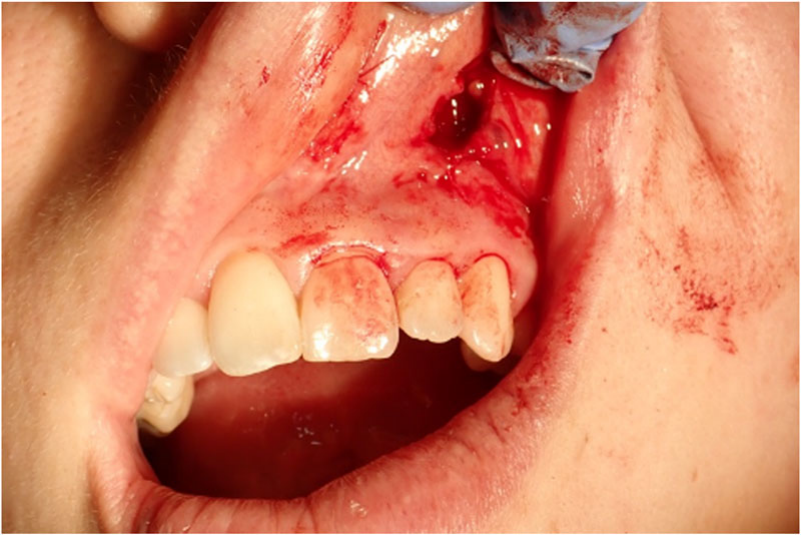
Patient 2. The picture displays the upper gingival mucosa after the lesion was removed.

**Figure 8:**
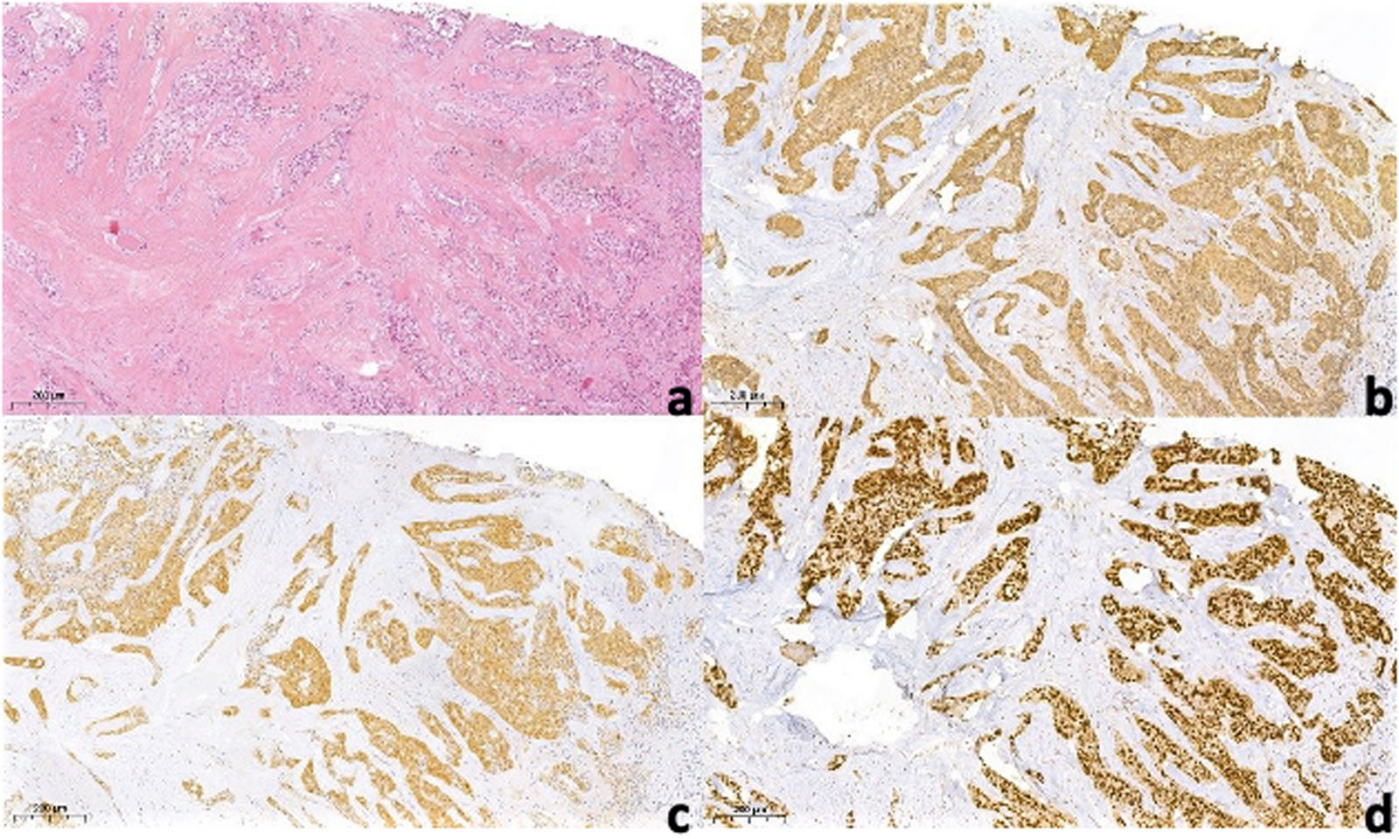
Secretory carcinoma of salivary glands. The neoplastic cells are arranged in solid nested and in ribbon and are dispersed in a prominent fibrous stroma (H&E, 10×) (a). Neoplastic cells show strong positive expression of mammaglobin (b), S‐100 (c) and TRK (d).

In the same month, surgery was conducted to remove the nodule with a reconstructive flap after plexus local anesthetic with 0.3 cc Articaine and vasoconstrictor. Safety margins of 4 mm were kept. The vestibular connective tissue and the bone plane to which the nodule is related appear to be cleavable, without invasion of the surrounding tissues (Figures 5, 6, and 7). A SC reaching the resection margins, close to a minor salivary glands, was determined according to the final histological study. Immunohistochemical analysis showed positivity of S100, mammaglobin, NTRK, and GATA3 and negativity for CD117; the proliferation index was 4%. FISH analysis was performed to confirm the diagnosis. In 95% of the cells examined, in fact, there was the rearrangement of NTRK3 gene (Figure 8).

#### 2.2.3 Outcome and Follow‐Up

**Figure 9:**
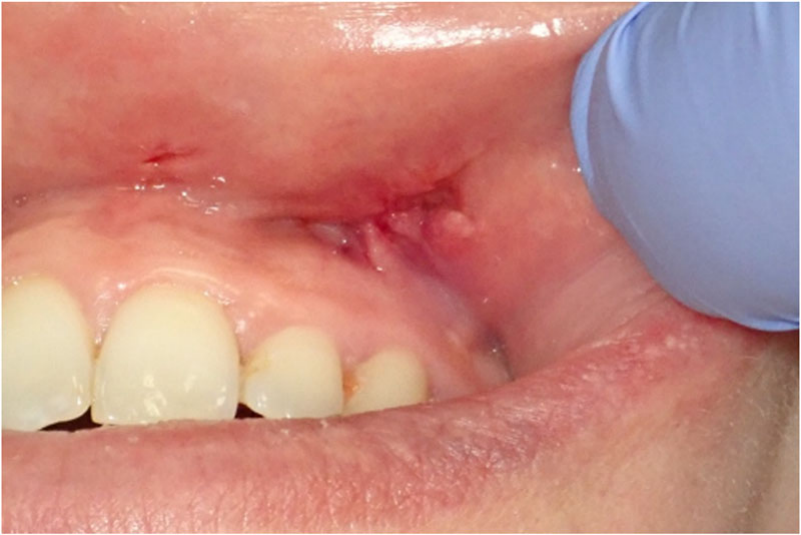
Patient 2's condition during the outpatient follow‐up session a month later is depicted in the figure.

**Figure 10:**
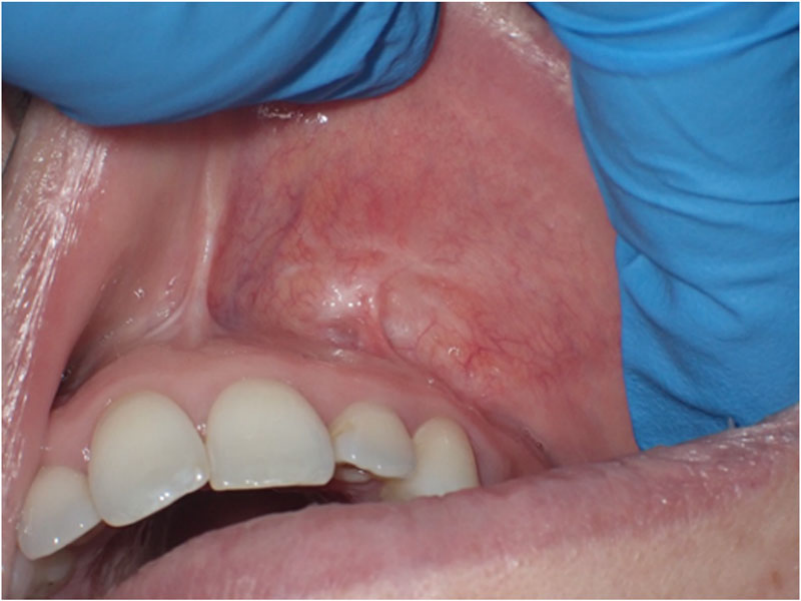
At the 6‐month follow‐up outpatient examination, patient 2 had a fibrotic scar.

**Figure 11:**
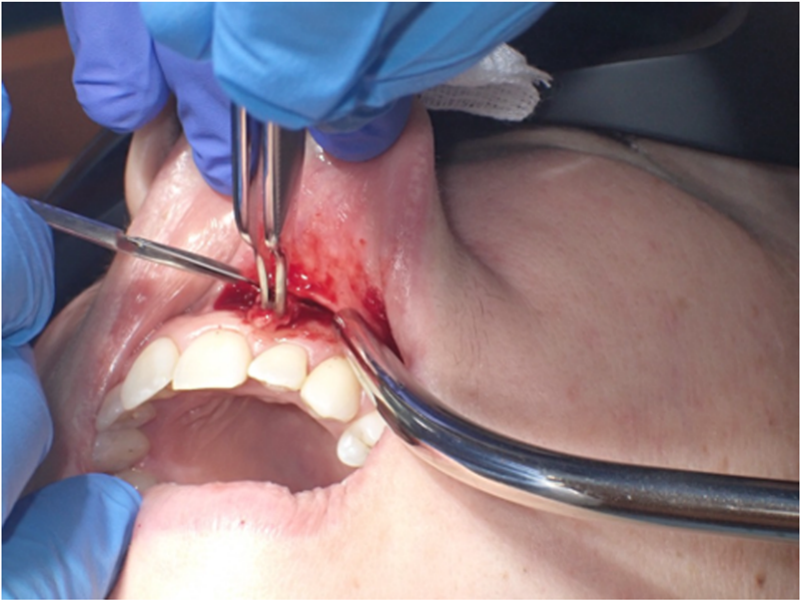
Patient 2. Intraoperative picture of the fibrotic scar excision.

**Figure 12:**
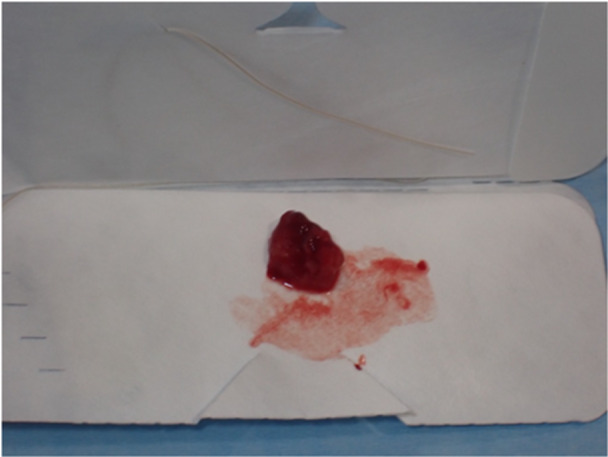
Patient 2. Fibrotic lesion surgically removed.

After consulting with the patient, it was agreed to conduct regular imaging monitoring because the tumor margins were described as “close” in the final histopathology report, initially avoiding the need for revision surgery. A month following surgery, the postoperative consult reveals satisfactory surgical results (Figure 9). The patient is still being followed up and has had several outpatient examinations with negative facial and neck MRIs, every 3 or 6 months. Clinically and radiologically, there are no signs of disease recurrence. After 6 months, it was planned for reoperation due to the palpatory discovery of suspected cicatricial fibrosis, decision that was also reinforced by the histological study results, which showed “close” margins, and the histological findings, removing the scar and widening of the resection margins, so a tiny scarred fibrous region adhering to the bone plane is removed (Figures 10, 11, and 12). The material removed is submitted for a final histological investigation, which reveals fibromuscular tissue free from neoplasia.

## 3 Discussion

The review with the highest number of cases report 659 instances of SC of the salivary glands, 14.3% of which arising in minor salivary glands (Silver et al. 2021). This case report reveals two more cases of SC of the minor salivary glands, increasing the growing body of evidence.

SCs are mainly low‐grade salivary gland carcinomas marked by strong similarities to mammary gland SC, both tumor types are triple (ER/PR/Her‐2) negative (Jackson et al. 2017) and bring ETV6‐NTRK3 gene fusion, according to data currently available. SCs have been included in the WHO Classification of Head and Neck tumors in 2017 (Sun et al. 2021).

SC presents as a circumscribed mass composed of microcystic, tubular, and solid structures with a bubbly secretory material within microcystic and tubular spaces (Venkat et al. 2021). These secretions stain with Periodic acid–Schiff (PAS) and Alcian blue. The neoplastic cells in SC have a considerable amount of vacuolated or granular, eosinophilic cytoplasm, large vesicular nuclei with finely granular chromatin and a single, small, centrally located nucleolus (Hua et al. 2021). Cytokeratin 7, cytokeratin 18, S100, vimentin, mammaglobin, MUC4, and STAT5a are important immunohistochemical stains for SC, which are frequently strong and diffuse (Roy et al. 2018; Skálová et al. 2010; Bishop et al. 2013; Venkat et al. 2021). Neoplastic cells are conversely negative for P63, DOG‐1, calponin, cytokeratin 6, cytokeratin 14, and smooth muscle actin (Baghai et al. 2017; Ni et al. 2016). These characteristics aid in differential diagnostic procedure (Skalova 2013). ACC is the most prevalent tumor in the parotid gland that is frequently misinterpreted with this condition (Sun et al. 2019). The analogies between SC and ACC are remarkable. PAS‐positive globules can be seen in both SC and ACC; however, PAS‐positive secretory zymogen cytoplasmic granules are specific to ACC (Roy et al. 2018; WHO Classification of Tumours Editorial Board 2023). Furthermore, ACC also has cytological and structural variety, as it is made up of a mix of serous acinar, intercalated duct‐like, hobnail, vacuolated, transparent, and nonspecific glandular cells that are arranged in solid/lobular, microcystic, papillary‐cystic, and follicular development patterns (Seethala and Stenman 2017). SCs, on the other hand, are architecturally homogeneous, with secretory material in lumina and microcystic and slightly dilated glandular spaces (Skálová et al. 2010) with intraluminal eosinophilic secretions that are rare or absent in ACC (Hua et al. 2021). Negative DOG1 with positive S‐100 and mammaglobin staining can be useful in the differential diagnosis between SC and ACC (Baghai et al. 2017; WHO Classification of Tumours Editorial Board 2023; Venkat et al. 2021). Some authors claim that immunohistochemistry positivity for mammaglobin and S‐100 combined with DOG‐1 negative can lead to diagnosis of SC of the salivary glands (Pinto et al. 2014). However, it is important to underline that positivity for mammaglobin and S100 are not specific to SC. In most cases, the proliferation index (Ki‐67) is low (less than 5%), as confirmed also by our cases (Pinto et al. 2014).

The translocation t (12; 15) (p13; q25), which results in a chimeric tyrosine kinase ETV6‐NTRK3, is a distinctive feature of SC of the salivary glands and the gold standard for its diagnosis, in fact it has a prevalence of more than 90% (Min et al. 2021). The same rearrangement is also present in SC of the breast. Other salivary gland tumors do not have this rearrangement (Bissinger et al. 2017). The protein encoded by ETS variant 6 (ETV6) gene belongs to ETS family transcription factor and the gene is located on chromosome 12; the neutrophic tyrosine kinase receptor 3 (NTRK3) encodes for tropomyosin receptor kinase C (TRKC) and the gene is located on chromosome 5 (Bissinger et al. 2017). TRKC is a proto‐oncogene involved in some biological processes such as neuronal survival, differentiation, and plasticity (Bissinger et al. 2017). FISH is the preferred method for identifying this translocation (Sun et al. 2021). The fusion gene produces a chimeric tyrosine kinase with transformational potential and a function in carcinogenesis (Hamada et al. 2004; Griffith et al. 2013): the fusion gene's product might cause angiogenesis through high level of vascular endothelial growth factor and tissue hemorrhage with hemosiderin deposition (Skálová et al. 2022). The ETV6–NTRK3 translocation has previously been found in congenital mesoblastic nephroma, infantile fibrous sarcoma, and myelogenous leukemia (Baghai et al. 2017; Csanyi‐Bastien et al. 2021; Boliere et al. 2019). FISH and in general molecular analysis are quite expensive and not all laboratories have appropriate instrumentations and qualified personnel to perform them (Bishop 2013). Moreover, they can require until 10 days for a correct result. For all these reasons, a relatively new immunohistochemical analysis was introduced in the last few years thanks to the development of the NTRK antibody (Bissinger et al. 2017). In fact, immunohistochemistry is available in every laboratory, routinely used, cheaper, and rapid (Bissinger et al. 2017). Authors report that NTRK IHC has a sensitivity of 78.3% and a specificity of 100% in detecting SCs with NTRK3 fusions when only nuclear staining is considered (Bissinger et al. 2017). Our cases confirm these data: in both patients more than 95% of neoplastic cells show intensive nuclear staining for NTRK immunostain.

ETV6 rearrangement with non‐NTRK3 fusion partners has been described; sometimes partners are identified (ETV6‐RET or ETV6‐MET are two examples) (Kuwabara et al. 2018), and sometimes they are unknown (ETV6‐X) (Knezevich et al. 1998; Eguchi et al. 1999; Ito et al. 2015). Ito et al. (2015) report two cases of SC with ETV6‐X in which both tumors have an histology of stromal fibrosis and invasive characteristics of perineural or vascular tumor invasion; this might suggest a correlation between the fusion partner and possible undesirable tumor characteristics. Obviously, in these cases, pan‐TRK immunohistochemistry is not helpful for diagnosis.

SC is a low‐grade malignant tumor with a good prognosis and a low recurrence rate that occasionally spreads to local lymph nodes and distant tissues. Nevertheless, SC can develop into a high‐grade malignant tumor and/or can have an aggressive behavior. SCs of salivary glands seldom infiltrates nearby structures, and perineural and vascular invasion are uncommon (Jackson et al. 2017; Griffith et al. 2013). Silver et al. (2021) review shows that 40 patients out of 659 reported cases presented with locoregional metastasis (6.1%) and 6 cases with distant metastasis (0.9%). Recurrence was discovered in 48 instances (7.3%) and 14 deaths were recorded. SCs have a 15% chance of recurrence locally, especially if they are not entirely removed (Skálová et al. 2010).

Treatment choices for salivary gland SCs do not differ significantly from other histotypes of salivary gland tumors, according to the NCCN recommendations. In essence, surgical excision is the primary line of therapy, with lymph node dissection as an option if lymph nodes are also implicated (Venkat et al. 2021). When surgery was not radical or additional risk factors were present (intermediate or high grade tumors, neural/perineural invasion, lymph node metastases, lymphatic or vascular invasion, or T3 or T4a tumors), adjuvant radiation therapy was suggested. The only substantial treatment variation in the NCCN recommendations for salivary gland SCs is in situations of recurrent tumors or tumors that cannot be surgically treated or metastatic ones. In these cases, if the translocation of the gene encoding NTRK is present, the use of TRK inhibitors (e.g., larotrectinib or entrectinib is considered useful) (Petersson et al. 2020; Skálová et al. 2016; Drilon et al. 2016). In these cases, the use of IHC to detect patients that are eligible for therapies can help reduce timing to start treatment, even if in some cases the use of molecular analysis remains mandatory (Bissinger et al. 2017; Bishop 2013).

## 4 Conclusions

**Figure 13:**
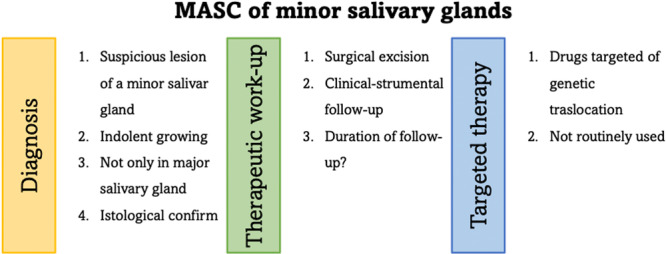
Graphic table of content.

In this study, we focused on two rare cases of SC of the minor salivary glands, which were located on the hard palate and in the vestibular mucosa, respectively, explaining the diagnostic and therapeutic workup (Figure 13). Both patients were treated surgically, and there were no evidence of illness recurrence after a year of follow‐up, any neck dissection wasn't conducted. In any event, because there is still no clear data on the length of the follow‐up, these patients' outpatient surveillance will be extended. There are no clinical diagnostic criteria for the diagnosis of SC; to present, only histological examination of the surgical specimen and the help of immunohistochemical studies and molecular analysis may bring to a correct diagnosis. We should not forget that the differential diagnosis especially with ACC could be very challenging. This implies that careful histology examinations are necessary. This study aims not only to emphasize the necessity of a precise diagnosis of this rare tumor, but also to improve the amount of scientific knowledge available so that more and more targeted therapies may be developed.

## Author Contributions

S.A., E.S., M.T. and A.G. were involved in the conceptualization of the case report. G.A. and D.F. focused on pathological details. S.A., A.L., G.A. and M.T. were involved in validation and collection of cases. E.S. and M.T. were involved in data curation. E.S. and D.F. were involved in writing – original draft preparation. S.B., M.T., A.L. and G.G were involved in writing – review and editing. All authors have read and agreed to the published version of the manuscript.

## Ethics Statement

The present article has been approved by the Ethics Committee of San Raffaele Hospital (20210422), in accordance with the Declaration of Helsinki.

## Consent

Written informed consent was obtained from the patient to publish this report in accordance with the journal's patient consent policy. All patients gave their consent.

## Conflicts of Interest

The authors declare no conflicts of interest.

## Data Availability

Data sharing is not applicable to this article as no new data were created or analyzed in this study. Data sharing is not applicable to this article, as no datasets were generated or analyzed during the current study.
